# Study on the In Vitro and In Vivo Antioxidant Activity and Potential Mechanism of *Polygonum viviparum* L.

**DOI:** 10.3390/antiox14010041

**Published:** 2025-01-01

**Authors:** Zhen Yang, Jingyuan Man, Haoyu Liu, Di Wu, Qiangwen Gu, Hongjuan Zhang, Yu Liu, Dan Shao, Baocheng Hao, Shengyi Wang

**Affiliations:** 1 Key Laboratory of New Animal Drug Project, Gansu Province, Key Laboratory of Veterinary Pharmaceutical Development, Ministry of Agriculture and Rural Affairs, Lanzhou Institute of Husbandry and Pharmaceutical Sciences of Chinese Academy of Agriculture Sciences, Lanzhou 730050, China; yangzhen01@caas.cn (Z.Y.); hk1013264544@163.com (J.M.); lhy000503@163.com (H.L.); wudi@caas.cn (D.W.); zhanghongjuan@caas.cn (H.Z.); liuyu@caas.cn (Y.L.); shaodan@caas.cn (D.S.); 2 College of Veterinary Medicine, Gansu Agricultural University, Lanzhou 730070, China; 3 Animal Husbandry and Veterinary Workstation, Heli Town, Gaotai County, Zhangye 734000, China; gsguxiangwen@126.com

**Keywords:** *Polygonum viviparum* L., oxidative stress, ESR1/MMP2 signaling pathway, JAK2/STAT3/ BCL2L1 signaling pathway

## Abstract

Oxidative stress refers to the phenomenon in which the redox balance of the body is disrupted in response to stimuli, leading to an excessive accumulation of reactive oxygen species in vivo, which can lead to a variety of diseases. In contrast to artificial antioxidants, whose safety is controversial, natural antioxidants, which are widely available, pharmacologically active, and have little toxic side effects, are expected to be candidates for the treatment of oxidative stress-related diseases. *Polygonum viviparum* L. (PV) is a natural herbal medicine with antioxidant properties and is used as a traditional medicine in the Tibetan Plateau region. However, there are few studies that have focused on its antioxidant activity and mechanism of action in vitro and in vivo. Therefore, the present study firstly demonstrated that PV could exert good in vitro antioxidant effects by scavenging DPPH radicals and inhibiting the production of hydroxyl radicals through in vitro experiments. Secondly, PV was proven to attenuate the effects of oxidative stress on body weight gain and thymus development by establishing the Senna leaf-induced diarrhea model in rats, as well as to increase the activity of antioxidant enzymes and the content of non-enzymatic antioxidants in the intestinal tract and to enhance the rats’ own antioxidant defenses, to mitigate the oxidative damage caused by diarrhea. Subsequently, the application of the cellular oxidative stress model evidenced that PV could play a protective role against cellular oxidative stress by inhibiting the overaccumulation of ROS in macrophages. Furthermore, the candidate antioxidant targets of PV were analyzed and screened using a comprehensive network pharmacology method, and their expression were then examined at the mRNA level and protein level. Our results suggest that PV may protect against H_2_O_2_-induced oxidative damage in macrophages by activating BCL2L1 and inhibiting ESR1, JAK2/STAT3, and MMP2. These findings open new perspectives on the antioxidant mechanism of PV and the prospect of developing it as a novel natural antioxidant drug.

## 1. Introduction

Oxidative stress refers to the phenomenon in which the amount of reactive oxygen species (ROS) and reactive nitrogen species (RNS) free radicals in cells and tissues exceed the range that can be scavenged by the body’s own antioxidant defense system when the body is subjected to a stimulus, resulting in an imbalance of redox balance in the body [1,2]. ROS free radicals such as hydrogen peroxide (H_2_O_2_), hydroxyl radicals (OH·), and superoxide anion (O_2_^−^) generated by cellular activities accumulate in excess under oxidative stress, causing damage to biological macromolecules, such as proteins, lipids, and DNA, which is one of the culprits of many diseases in living organisms [3]. Studies have reported that antioxidant supplementation can effectively maintain cellular ROS homeostasis through endogenous pathways and restore redox balance in the body [4,5]. As a result, a great deal of effort has been devoted to better research on antioxidants. Different from artificial antioxidants, whose safety is subject to a great deal of controversy, antioxidants screened from natural products with potential therapeutic effects, such as plant polysaccharides, flavonoids, polyphenols, etc., are characterized by a wide range of sources, high antioxidant capacity, and low toxicity and side effects [6], and have, therefore, become a research hotspot for antioxidant development in recent years.

Alpine meadows are important livestock production bases on the Qinghai–Tibetan Plateau, with rich grass species and high nutritional value. Among them, *Polygonum viviparum* L. (PV) is the dominant or subdominant species in the alpine meadow ecosystem [7,8]. It is recorded in Jingzhu Materia Medica that PV tastes sweet, astringent, sour, and has the effect of preventing diarrhea and alleviating intestinal cold pain, etc. Its stems and leaves can be used as high-quality fodder, and its roots are rich in starch; in addition to being used as medicinal materials, it can also be used for brewing with fruits [9]. Modern studies have shown that PV contains a variety of chemical components including flavonoids, flavonoid glycosides, organic acids, volatile oils, tannins, polysaccharides, and trace elements [8]. In addition, due to its various pharmacological activities such as antioxidant, anti-tumor, and antibacterial properties, PV has shown promising effects in the treatment of diseases including bronchitis, hemorrhoids, ulcers, and vomiting [10,11]. However, only a few studies have been reported on the chemical composition and pharmacological activity of PV, and further studies are urgently needed. Antioxidant activity is one of the important pharmacological activities of PV, but there are few studies on its in vivo and in vivo antioxidant activity and mechanism of action. Therefore, in-depth research on the in vivo and in vivo antioxidant activity and mechanism of action of PV is of great significance to improve its economic and medicinal value.

In this study, methods of network pharmacology and molecular biology were combined to investigate the in vivo and in vivo antioxidative effect of PV and its potential mechanism. First, we evaluated the in vitro antioxidant capacity of PV using traditional in vitro antioxidant assays. Then, the in vivo antioxidant effects of PV were evaluated by monitoring body weight and thymus index and by determining the catalase (CAT), reduced glutathione (GSH), malonaldehyde (MDA), and superoxide dismutase (SOD) of intestinal tissues using the Senna leaf-induced diarrhea model in rats. In addition, the oxidative stress model of RAW264.7 cells induced by H_2_O_2_ was established to determine the intracellular antioxidant effects of PV by measuring the cellular ROS generation. Following this, a network pharmacology approach was applied to screen the active ingredients and antioxidant targets of PV, and further protein–protein interaction (PPI) and topology analyses were performed. Finally, RT-qPCR and Western blotting were used to validate the antioxidant targets of PV screened by network pharmacology at the mRNA level and protein level and to speculate the potential antioxidant mechanism of PV.

## 2. Materials and Methods

### 2.1. Materials and Reagents

The roots of PV were brought from Gansu Fuxinghou Biomedical Technology Co., Ltd. (Lanzhou, China). The specimen of PV (Specimen No. 20230115) was kept in Key Laboratory of Veterinary Pharmaceutical Development of Ministry of Agriculture, Lanzhou Institute of Husbandry and Pharmaceutical Sciences of CAAS (Lanzhou, China). Baitouweng oral liquid (BTW) was obtained from Shandong Yiyuan Pharmaceutical Co., Ltd. (Heze, China). The murine macrophage cell line (RAW 264.7 cells) was purchased from Cell Culture Center of the Chinese Academy of Sciences (Shanghai, China). Dulbecco’s modified Eagle’s medium (DMEM) high glucose and fetal bovine serum (FBS) were obtained from HyClone (Marlborough, MA, USA) and Gibcol Life Technology (Thermo Fisher, Waltham, MA, USA). Cell Counting Kit-8 (CCK-8) was acquired from Biosharp Life Sciences (Hefei, China). Phosphate-buffered saline (PBS), BCA protein concentration determination kit, catalase (CAT), reduced glutathione (GSH), malonaldehyde (MDA), and superoxide dismutase (SOD) detection kits were purchased from Solarbio Science & Technology Co., Ltd. (Beijing, China). A ROS assay kit was purchased from Nanjing Jiancheng Bioengineering Institute (Nanjing, China). Ethanol, salicylic acid, ferrous sulfate (FeSO_4_), hydrogen peroxide (H_2_O_2_), and other chemical reagents used in experiments were chromatographic-grade and obtained from local suppliers. 2,2-diphenyl-1-picrylhydrazyl (DPPH) was purchased from TCI (Shanghai) Development Co., Ltd. (Shanghai, China), and ethylene diamine tetraacetic acid disodium salt (EDTA-2Na) and ascorbic acid (Vc) were purchased from Sinopharm Chemical Reagent Co. (Shanghai, China). The Simply P total RNA extraction kit was provided by Bioer Technology Co., Ltd. (Hangzhou, China). TB Green Premix Ex Taq II and the PrimeScript RT reagent kit with gDNA eraser were obtained from Takara Bio (Shiga, Japan).

### 2.2. Preparation of PV

The roots of PV were air-dried and ground, and then the powder of PV was obtained after sieving by 200 mesh. A certain amount of powder was accurately weighed and immersed in ultrapure water for 30 min and then extracted by the ultrasonic extraction method. The amount of ultrapure water was 6–10 times the mass of PV powder; the extraction temperature was 40–60 °C; the extraction time was 60–120 min; the ultrasonic power was 400–600 W; and the extraction times were 3 times. The extracts of the 3 times were combined and filtered, decolorized using activated charcoal, and then deproteinized using the Sevage method. The extract was concentrated by rotary evaporation and then added with anhydrous ethanol solution to ethanol content of 95% for alcohol precipitation. The alcoholic precipitation solution was allowed to stand overnight at 4 °C. The supernatant of the alcohol precipitation solution was discarded, and the precipitate was centrifuged at 3000 rpm/min. The freeze-dried precipitate was PV extract.

### 2.3. Detection of In Vitro Antioxidant Activity

#### 2.3.1. DPPH Radical Scavenging Ability

The DPPH free radical scavenging activity of PV was measured and modified slightly according to reported procedure [12]. DPPH solution was prepared in 75% ethanol at a concentration of 1 mM. A total of 100 μL of various concentrations of PV or ascorbic acid (Vc) was mixed with 200 μL of DPPH solution in 96-wells plates. The mixture was allowed to stand in the dark at room temperature for 30 min, and the absorbance value at 517 nm was recorded with a spectrophotometer (Epoch Microplate Spectrophotometer, BioTek Instruments, Inc., Montpelier, VT, USA). The DPPH radical scavenging activity of PV was calculated by the following equation:

DPPH radical scavenging activity (%) = [1 − (A_1_ − A_2_)/A_0_] × 100%

where A_0_ was the absorbance of the control group (DPPH solution without sample). A_1_ was the absorbance of the test sample mixed with DPPH. A_2_ was the absorbance of the sample (75% ethanol without DPPH). Vc was used as the positive control.

#### 2.3.2. Hydroxyl Radical Scavenging Ability

The method used to measure hydroxyl radical scavenging ability in this study was modified based on previous reporting [13]. Briefly, 50 μL of 9 mM FeSO_4_ aqueous solution, 50 μL of 9 mM salicylic acid–ethanol solution, and 50 μL of 3.8 mM H_2_O_2_ were added successively in 96-wells plate that containing 50 μL of different concentrations of PV and Vc. The mixture was allowed to stand in the incubator at 37 °C for 30 min, and the absorbance value at 510 nm was recorded with a spectrophotometer (Epoch Microplate Spectrophotometer, BioTek Instruments, Inc., USA). The hydroxyl radical scavenging ability of PV was calculated by the following equation:

Hydroxyl radical scavenging activity (%) = [1 − (A_1_ − A_2_)/A_0_] × 100%

where A_0_ was the absorbance of the control group (distilled water instead of sample). A_1_ was the absorbance of the test sample mixed with the reaction solution. A_2_ was the absorbance of the sample (distilled water instead of H_2_O_2_). Vc was used as the positive control.

#### 2.3.3. Ferrous Ion Chelating Ability

The Ferrous ion chelating ability of PV was evaluated based on an earlier reported method with some modifications [14]. In brief, 5 μL of 4 mM FeCl_2_·H_2_O and 20 μL of 5 mM ferrozine were added successively in 96-wells plate containing 100 μL of different concentrations of PV and EDTA-2Na. After 75 μL of distilled water was added to each well, the mixture was placed at room temperature for 10 min. Following incubation, the absorbance was measured at 560 nm with a spectrophotometer (Epoch Microplate Spectrophotometer, BioTek Instruments, Inc., USA). The ferrous ion chelating ability of PV was calculated by the following equation:

Ferrous ion chelating ability (%) = [1 − (A_1_ − A_2_)/A_0_] × 100%

where A_0_ was the absorbance of the control group (distilled water instead of sample). A_1_ was the absorbance of the test sample mixed with the reaction solution. A_2_ was the absorbance of the sample (distilled water instead of ferrozine). EDTA-2Na was used as the positive control.

#### 2.3.4. Ferric Reducing Power

The ferric reducing ability of PV was determined according to the ferric reducing antioxidant power (FRAP) method described by Parolia with minor modification [15]. The reaction solution included 100 μL different dilutions of PV and Vc, 250 μL phosphate buffer (pH 6.6), and 250 μL potassium ferricyanide (1 wt %). After incubation in a water bath at 50 °C for 20 min, 250 μL of trichloroacetic acid solution (10 wt %) was added to the mixture and centrifuged at 4000 rpm for 10 min. A total of 50 μL of distilled water and FeCl_3_ (1 wt %) was mixed with 50 μL of supernatant in 96-wells plate, and the absorbance was measured at 700 nm with a spectrophotometer (Epoch Microplate Spectrophotometer, BioTek Instruments, Inc., USA). The ferric reducing antioxidant power of PV was calculated by the following equation:

Ferric reducing antioxidant power = A_1_ − A_2_

where A_1_ was the absorbance of the test group. A_2_ was the absorbance of all the reagents (distilled water instead of FeCl_3_ solution). Vc was used as the positive control.

### 2.4. Detection of In Vivo Antioxidant Activity

#### 2.4.1. Animals

Sprague Dawley (SD) rats (SPF grade) were provided by Lanzhou Veterinary Research Institute (Quality Certification of Laboratory Animals: SCXK 2020-0002, Lanzhou, China). The rats were housed in an SPF facility (22 ± 2 °C and 60 ± 5% humidity) with a 12 h/12 h light/dark cycle (lights on from 08:00 to 20:00). Drinking water and standard rat food were provided ad libitum. All the animal experiment procedures were performed strictly in compliance with the National Institutes of Health Guide for Care and Use of Laboratory Animals and approved by the Animal Ethics Committee of Lanzhou Institute of Husbandry and Pharmaceutical Sciences of CAAS (protocol code: 2022-004).

#### 2.4.2. Establishment of Diarrhea Model in Rats

After one week of acclimatization, rats were randomly divided into six groups (*n* = 6, 50/50 male and female), i.e., control, model, Baitouweng (BTW, 2.5 g/kg·bw), and three treatment groups of PV (PV-L, 0.3 g/kg·bw; PV-M, 0.9 g/kg·bw; PV-H, 1.8 g/kg·bw). Except for the normal group, which was given the same volume of distilled water by gavage, the rats in other groups were given a Senna decoction (1.5 g/kg·bw) by gavage for 7 consecutive days to establish the diarrhea model. On the 7th day of modeling, after 1 h of Senna gavage, the normal group and model group were given normal saline by gavage, and the rest of the groups were given the corresponding drugs by gavage for 3 consecutive days. All rats were weighted and then executed and dissected. The thymus and duodenum were collected. The organ index was calculated according to the following equation.

Organ index = (organ weight/body weight) × 100%

#### 2.4.3. Detection of Antioxidant Indexes in Intestinal Tissue

Intestinal catalase (CAT) and superoxide dismutase (SOD) activities and glutathione (GSH) and malondialdehyde (MDA) contents were determined using commercial kits according to the manufacturer’s instructions (Beijing Solarbio Science & Technology Co., Ltd., Beijing, China).

### 2.5. Detection of Intracellular Antioxidant Activity

#### 2.5.1. Cell Culture

The RAW264.7 macrophages were cultured in DMEM supplemented with 10% FBS at 37 °C in a fully humidified incubator containing 5% CO_2_. Cell passaging was performed by the conventional method when the cells grew into a dense monolayer.

#### 2.5.2. Cell Viability Assay

Cell viability was measured by the Cell Counting Kit-8 (CCK-8) assay according to a previous procedure with slight modifications [16], and the H_2_O_2_-induced oxidative stress model was established following the published method with some modifications [17]. The RAW264.7 cells were seeded in culture medium for 4 h into a 96-well plate, with 1 × 10^4^ per well. The cells were treated with DMEM containing different concentrations of PV for 24 h. The cell viability stimulated with PV under oxidative stress was treated as follows: the RAW264.7 cells were seeded in culture medium for 4 h into a 96-well plate, with 1 × 10^5^ per well, then treated with DMEM containing different concentrations of PV for 20 h. Next, the positive control group and PV-treated groups were exposed to H_2_O_2_ (400 μM) for 4 h. A total of 10 μL CCK-8 solution was added to each well, and the plate was incubated at 37 °C in an atmosphere containing 5% CO_2_ for 4 h. The optical density (OD) was recorded at 450 nm by using a spectrophotometer (Epoch Microplate Spectrophotometer, BioTek Instruments, Inc., USA). Cell viability was calculated according to the following formula:

Cell Viability (%) = [(OD_treatment_ − OD_blank_)/(OD_control_ − OD_blank_)] × 100%

#### 2.5.3. Detection of Cellular ROS Production

Cellular ROS production was measured by a dichlorodihydrofluorescein diacetate (DCFH-DA) assay, through a previously described procedure with modifications [18]. For evaluation of ROS production, RAW264.7 cells were seeded in the confocal dish and cultured at 37 °C for 24 h. After different treatment, cells were incubated with DCFH-DA (10μM, Jiancheng Bioengineering Institute, Nanjing, China) in dark for 30 min at 37 °C and then washed twice by PBS. Fluorescence was observed by confocal microscopy. The mean fluorescent intensity of treatment group was normalized to that of the control group.

### 2.6. Network Pharmacology Analysis

#### 2.6.1. Active Ingredients of PV

All chemical constituents of PV obtained from published literature were screened under the following conditions: (1) ingredients with oral bioavailability (OB) ≥ 30% and drug-likeness (DL) ≥ 0.18; (2) the ingredient has been reported to possess antioxidant activity. The SMILE structures of screened ingredients and their targets used for subsequent analysis were obtained through PubChem database (https://pubchem.ncbi.nlm.nih.gov/, accessed on 12 July 2023) and SwissTarget Prediction database (http://www.swisstargetprediction.ch/, accessed on 12 July 2023), respectively.

#### 2.6.2. Common Targets Between Drug and Disease

The GeneCards database (https://www.genecards.org/, accessed on 12 July 2023) was used to search disease-related genes with the keyword “antioxidation”. The targets of the bioactive ingredients of PV were mapped to the target genes related to “antioxidation” to obtain the common target genes through the online tool “jvenn” (http://jvenn.toulouse.inra.fr/app/example.html, accessed on 12 July 2023).

#### 2.6.3. Protein–Protein Interaction Network

The protein–protein interaction (PPI) network was constructed through the STRING database (https://string-db.org/, accessed on 13 July 2023) according to the overlap of predicted targets of PV and antioxidation related targets to further elucidate the potential antioxidant mechanism of PV. The protein interaction information including the node degree value was obtained with the conditions set as “Homo sapiens”, “Hide disconnected nodes in the network”, and “Minimum required interaction score = 0.4”. The PPI network was visualized by Cytoscape 3.7.2 software, and the topological properties of each node and the selected hub gene were analyzed by the “network Analysis” plug-in based on the obtained node degree values.

#### 2.6.4. GO and KEGG Pathway Enrichment

To analyze the biological pathways of genes in the PPI network, the DAVID database (https://david.ncifcrf.gov/home.jsp, accessed on 13 July 2023) was used to analyze the enrichment of Gene ontology (GO) in biological function/process (BP), cellular component (CC), and molecular function (MF), as well as the Kyoto Encyclopedia of Genes and Genomes (KEGG) pathway, with the condition set to “Homo sapiens” (adjusted to *p* < 0.05). The R-based online graphing tools “Bioinformatics” (https://www.bioinformatics.com.cn, accessed on 13 July 2023) and “ChiPlot” (https://www.chiplot.online/, accessed on 13 July 2023) were used to visualize information of GO enrichment and KEGG pathway enrichment.

### 2.7. Detection of mRNA Expression

Total RNA of cells in different treatment groups was extracted using the Simply P Total RNA Extraction Kit (Bioflux, Hangzhou, China) and reverse-transcribed into cDNA using the PrimeScript™ RT reagent Kit with gDNA Eraser (Perfect Real Time) (Takara, Shiga, Japan). Quantitative real-time PCR was performed using QuantStudio (Thermo Fisher, USA) with TB Green^®^ Premix Ex Taq™ II (Takara, Japan). The fold changes in relative mRNA expression were calculated according to 2^−ΔΔCT^ by comparing the β-actin normalized threshold cycle numbers. The sequences designed for qRT-PCR analysis are listed in Table 1.

### 2.8. Western Blotting Analysis

RIPA lysis buffer containing PMSF was added to extract cell protein. The protein concentration was measured by a bicinchoninic acid protein assay kit. The protein sample was separated by 10% SDS-PAGE and then transferred to NC membranes. After the membrane was washed by TBST buffer and blocked with QuickBlock™ blocking buffer for 15 min, it was incubated with the corresponding antibody solution (1:1000) at 4 °C overnight. Afterward, the NC membrane was incubated with the secondary antibody at 37 °C for 1 h. Finally, the membrane was exposed to the ECL Western blotting detection reagent (Thermo Scientific, Waltham, MA, USA) for detecting chemiluminescence positive signals. Protein band images were scanned, and their integrated absorbance (IA) was analyzed by ImageJ 1.53e software. To eliminate the difference in protein loading, the relative level of the target protein was normalized to β-actin (target protein IA/β-actin IA).

### 2.9. Statistical Analysis

Statistical analysis was performed using SPSS 26.0 software for Windows (SPSS Inc., Chicago, IL, USA). The experimental results were expressed as the mean ± standard deviation (SD) of three independent experiments. Significant differences were evaluated by the one-way analysis of variance (ANOVA), followed by LSD multiple comparison. *p* < 0.05 was considered statistically significant.

## 3. Results

### 3.1. In Vitro Antioxidant Activity of PV

ROS free radical-mediated oxidative stress damage to vital substances such as proteins, lipids, and DNA has been shown to be closely related to the occurrence of various diseases in the organism. Therefore, in this study, we first evaluated the in vitro antioxidant activity of PV by detecting its ability to inhibit DPPH radicals and hydroxyl radicals, as well as its iron ion chelating ability and iron reducing ability.

**Figure 1:**
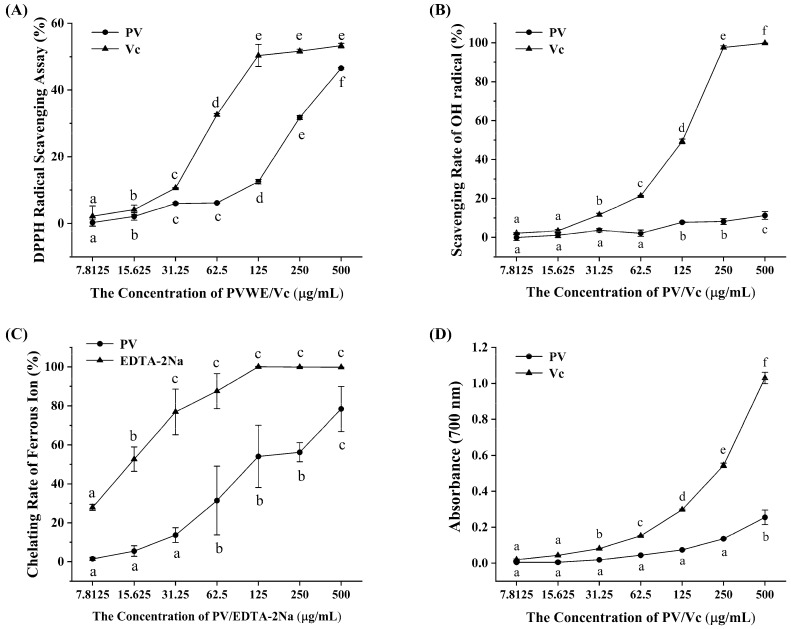
In vitro antioxidant activity of PV. (**A**) Scavenging activity of PV on DPPH free radicals. (**B**) Scavenging activity of PV on OH radicals. (**C**) Ferrous ion chelating ability of PV. (**D**) Ferric reducing power of PV. The results were expressed as the mean ± standard deviation of three independent experiments. The significant differences (*p* < 0.05) in the same sample were indicated by different letters.

DPPH is a stable free radical, which can be reduced to yellow diphenyl-picrylhydrazine after accepting an electron or hydrogen radical. Therefore, the scavenging capacity of an antioxidant for DPPH radicals can be determined based on the degree of decrease in absorbance it causes [19]. This assay is widely used to assess the total radical scavenging capacity of substances. As shown in Figure 1A, in general, both PV and Vc showed strong scavenging ability for DPPH radicals and enhanced with increasing sample concentration. The DPPH scavenging ability of both Vc and PV was weak in the concentration range of 7.8125 to 31.25 μg/mL. When the concentration of Vc was greater than 31.25 μg/mL, its ability to scavenge DPPH radicals increased sharply with increasing concentration, and this increasing trend leveled off when its concentration was higher than 125 μg/mL. The ability of PV to scavenge DPPH radicals followed a similar trend to Vc, with a dramatic increase in its ability to scavenge DPPH radicals when the concentration of PV was greater than 62.5 μg/mL. In addition, both Vc and PV had close to a 50% DPPH radical scavenging effect when their concentration reached 500 μg/mL.

The Fenton reaction has an accelerating effect on the lipid peroxidation chain reaction in addition to the production of hydroxyl radicals [20]. Hydroxyl radicals produced by the Fenton reaction are one of the most active free radicals in the organism, and they have the ability to react with all biomolecules in the cell [21]. In the present study, we evaluated the scavenging capacity of PV for hydroxyl radicals generated by the Fenton reaction. As shown in Figure 1B, the scavenging ability of Vc and PV for hydroxyl radicals increased with their concentrations, but PV showed a less pronounced trend of enhancement and exhibited a weaker hydroxyl radical scavenging ability. When the concentration of Vc reached 250 μg/mL, it was effective in scavenging nearly 100% of hydroxyl radicals. In contrast, PV was less than 20% effective in scavenging at all concentrations.

In addition to the direct scavenging of hydroxyl radicals, antioxidants can inhibit the Fenton reaction by chelating ferrous ions and reducing the production of hydroxyl radicals, thus, playing an antioxidant role [22]. Therefore, the chelating ability of PV on ferrous ions was evaluated in this study using EDTA-2Na as a positive control. The results in Figure 1C show that PV has a strong ferrous ion chelating ability, which is enhanced with the increase in sample concentration. When the concentration of EDTA-2Na was 125 μg/mL, its chelating ratio of ferrous ions reached 100%, while the chelation ratio of PV to ferrous ions was only about 50% at this concentration. When the concentration of PV was 500 μg/mL, its chelation ratio of ferrous ion was close to 80%. Based on the above results, it can be seen that PV mainly inhibits the generation of hydroxyl radicals by chelating free ferrous ions, thus, exerting its antioxidant effect.

The Fe^3+^ in K_3_[Fe (CN)_6_] was reduced to Fe^2+^ by substances with iron reducing capacity and then reacted with ferric chloride to form a complex with maximum absorption at 700 nm. The ability of PV to reduce Fe^3+^ to Fe^2+^ can be evaluated by measuring the absorbance at 700 nm [23,24]. As can be seen from Figure 1D, the iron reducing ability of both Vc and PV showed a certain dose-dependent trend, but the iron reducing ability of PV was significantly weaker than that of Vc. In addition, when the concentration of PV was lower than 250 μg/mL, there was no significant difference in the iron reducing capacity of different concentrations of PV (*p* > 0.05). When the concentration of PV was increased to 500 μg/mL the iron reducing capacity increased significantly compared to that of other concentrations (*p* < 0.05).

### 3.2. In Vivo Antioxidant Activity of PV

#### 3.2.1. Effect of PV on Body Weight and Thymus Index of Diarrhea Rats

**Figure 2:**
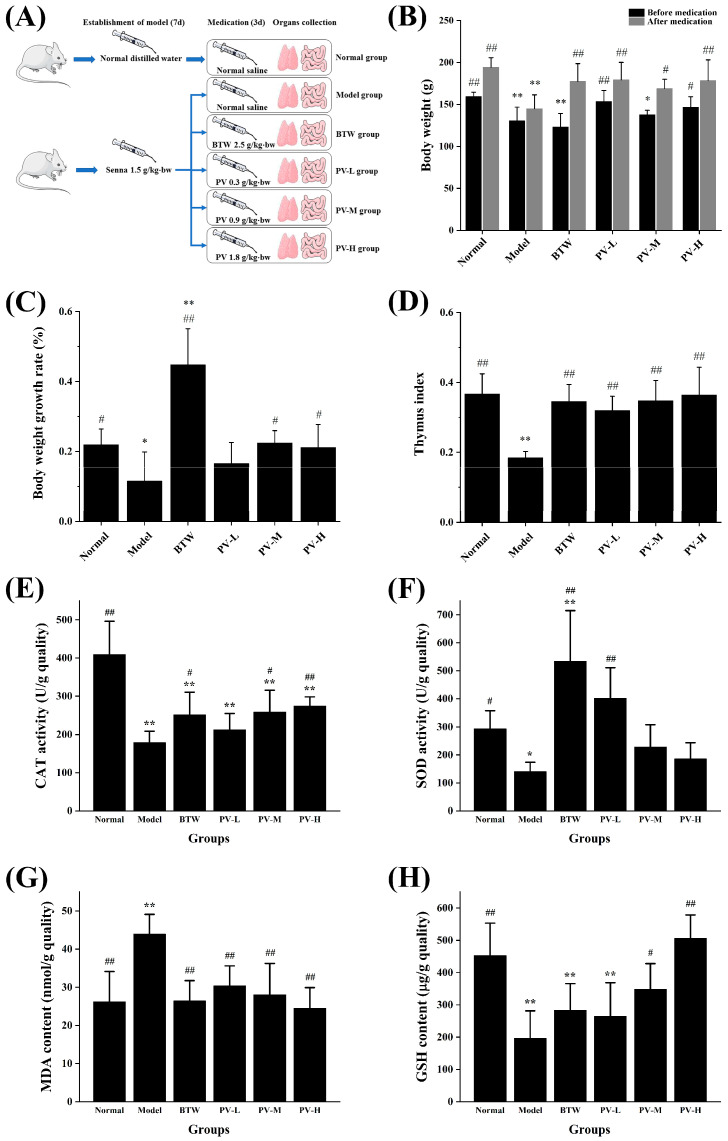
In vivo antioxidant activity of PV. (**A**) Graphical abstract of animal experiment. (**B**) Body weight changes in the different treatment groups. (**C**) Body weight growth rates in the different treatment groups. (**D**) Effect of PV on thymus index of SD rats. (**E**) Effect of PV on the activity of CAT in intestinal tissues of SD rats. (**F**) Effect of PV on the activity of SOD in intestinal tissues of SD rats. (**G**) Effect of PV on the content of MDA in intestinal tissues of SD rats. (**H**) Effect of PV on the content of GSH in intestinal tissues of SD rats. The results were expressed as the mean ± standard deviation of three independent experiments. * *p* < 0.05, ** *p* < 0.01, compared with those in the normal control group; # *p* < 0.05, ## *p* < 0.01, compared with those in the model group.

Changes in body mass can reflect the health status of rats to a certain extent [25]. In order to evaluate the anti-oxidative stress effect of PV in vivo, the present study first analyzed the changes in body weight and body weight growth rate of rats in each group before and after treatment. Figure 2B showed the changes in body weight of rats in each group. From the results, it can be seen that the body weights of rats in the control group and three doses of PV treatment group were significantly higher compared with the model group (*p* < 0.05). There was no significant difference in the body weight of the rats treated with three doses of PV compared to the control group (*p* > 0.05). The changes in body weight growth rate of rats in each group before and after drug administration were shown in Figure 2C. The body weight growth rate of rats in the model group was significantly lower than that of the control group (*p* < 0.05), and the body weight growth rate of rats in the medium- and high-dose groups of PV was significantly higher than that of the model group (*p* < 0.05) and not significantly different from that of the control group (*p* > 0.05). The above results suggested that PV has an ameliorating effect on the slow weight gain of rats with Senna-induced diarrhea.

The thymus is one of the main immune organs, which is involved in cellular and humoral immunity of the organism [26]. In the presence of oxidative damage in the body, the quality and morphological structure of thymus tissue may undergo certain changes, mainly manifested as a reduction in quality [27]. Therefore, changes in thymus index can reflect the level of oxidative damage in the organism to some extent [28]. The effects of PV on the level of oxidative damage in rats with Senna-induced diarrhea were investigated in this paper by analyzing the thymus index of each group of rats after drug intervention. As shown in Figure 2D, the thymus index of rats in the model group was lower than that of the control group, and the difference was extremely significant (*p* < 0.01). In contrast, the thymus indexes of the three-dose groups of PV were significantly higher compared with that of the model group (*p* < 0.01) and showed a dose-dependent trend. In addition, there was no significant difference in the thymus index of the BTW group and the three doses of PV group compared with the blank control group (*p* > 0.05). It can be concluded that PV can promote the development of the thymus in rats with diarrhea, delay thymus atrophy, and alleviate oxidative damage in rats.

#### 3.2.2. Effect of PV on Antioxidant Enzyme Activity of Diarrhea Rats

CAT controls the abundance of the cell signaling molecule, hydrogen peroxide, by catalyzing its decomposition. As a core antioxidant enzyme, CAT plays an important role in the antioxidant network of organisms [29]. CAT activity in intestinal tissues of rats in different treatment groups was shown in Figure 2E. Compared with control group, the CAT activity in intestinal tissue of the model group was significantly decreased (*p* < 0.01). There was no significant difference in CAT activity in the intestinal tissue of rats in the low-dose PV group compared with the model group (*p* > 0.05), whereas that of the medium- and high-dose groups was significantly higher (*p* < 0.05). SOD is the first line of defense against ROS-mediated oxidative damage. As a metalloenzyme, SOD catalyzes superoxide anion disproportionation to produce H_2_O_2_ and O_2_ through the Fenton reaction in the presence of Fe^2+^, thereby reducing the potential harm of superoxide anions [30]. Figure 2F showed the SOD activity in the intestinal tissue of rats in each treatment group, and in the model group it was significantly lower than the control group (*p* < 0.05). There was no significant difference in SOD activity in the intestinal tissue of rats in all PV dose groups compared with the control group (*p* > 0.05), but in the low-dose PV group it was significantly higher than the model group (*p* < 0.01). Furthermore, the SOD activities in intestinal tissue of rats in both medium- and high-dose PV groups were higher than that in model group, but the difference was not significant (*p* > 0.05).

MDA is one of the lipid peroxides generated by the action of ROS on unsaturated fatty acids in lipids, and as a lipid peroxidation marker in vivo, its content can reflect the degree of oxidative damage in the body [31,32]. As shown in Figure 2G, the MDA content in intestinal tissues of rats in the model group was significantly higher than that of the control group (*p* < 0.01). In contrast, the MDA contents in intestinal tissues of the three doses of PV treatment groups were not significantly different from that of the control group (*p* > 0.05) and was significantly lower than that of the model group (*p* < 0.01), with a certain dose-dependent trend. GSH, a highly abundant tripeptide molecule glutathione, is a non-enzymatic antioxidant that controls redox homeostasis in mammalian cells. As a key component of the cellular redox system, GSH is not only an antioxidant with direct protective effects against cellular damage caused by free radicals and oxidants but also a cofactor for other antioxidant and detoxifying enzymes [33,34]. The GSH content in the intestinal tissues of rats in each treatment group was shown in Figure 2H. Compared with the control group, the GSH content in the intestinal tissues of rats in the model group was significantly lower (*p* < 0.01), while there was no significant difference with that in the PV medium- and high- dose groups (*p* > 0.05). In addition, the GSH content in the intestinal tissues of rats treated with medium and high doses of PV was significantly higher compared with that in the model group (*p* < 0.05).

### 3.3. Intracellular Antioxidant Activity of PV

#### 3.3.1. Effect of PV on the Viability of RAW264.7 Cells

**Figure 3:**
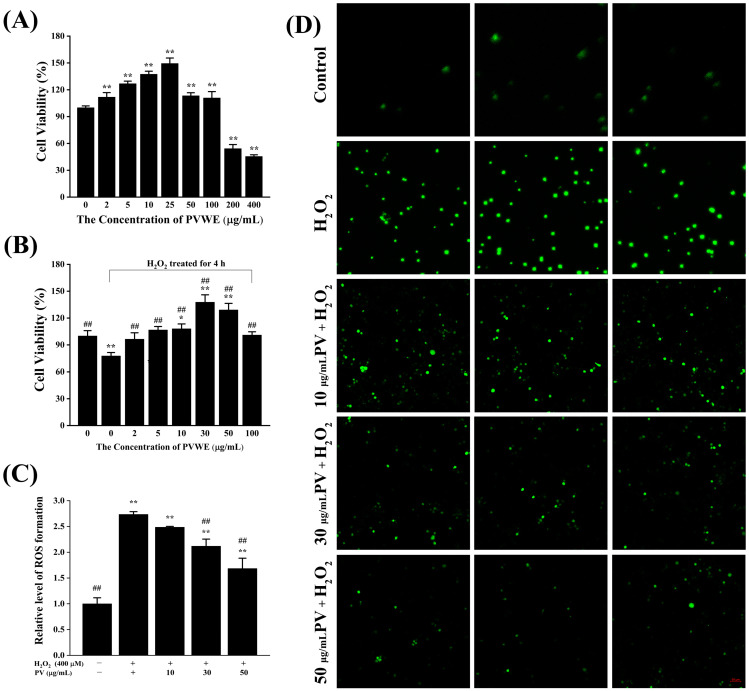
Intracellular antioxidant activity of PV. (**A**) The effect of PV on the viability of RAW264.7 cells. (**B**) The effect of PV on the viability of RAW264.7 cells under oxidative stress. (**C**) The relative levels of ROS generation. (**D**) Images of DCF fluorescence intensity. (Scale bar: 50 μm). The results were expressed as the mean ± standard deviation of three independent experiments. * *p* < 0.05, ** *p* < 0.01, compared with those in the normal control group; ## *p* < 0.01, compared with those in the model group.

The CCK-8 assay can effectively respond to the effect of drugs on cell proliferation by detecting the concentration of succinic dehydrogenase in cell mitochondria [35]. A CCK-8 assay was used in this study to determine the effect of different concentrations of PV on cell viability in order to screen the concentration of PV for use in subsequent experiments. As can be seen in Figure 3A, the viability of RAW264.7 cells first increased and then decreased as the concentration of PV increased. When the concentration of PV was 200 μg/mL, cell viability was significantly decreased compared with the control group (*p* < 0.01), which indicated that PV was cytotoxic at a concentration of 200 μg/mL. When the concentration of PV was in the range of 2–100 μg/mL, cell viability was significantly increased compared with the control group (*p* < 0.01). Among them, the highest cell viability was observed in cells treated with 25 μg/mL of PV. According to the above results, it was hypothesized that PV had a promoting effect on the proliferation of RAW264.7 cells in the concentration range of 2–100 μg/mL. Therefore, in this concentration range, concentrations of 2, 5, 10, 30, 50, and 100 μg/mL were selected for detecting the effect of PV on the cell viability of H_2_O_2_-induced macrophages. The results of the experiment were shown in Figure 3B; the cell viability of the H_2_O_2_ group was significantly decreased compared to the control group (*p* < 0.01). The cell viabilities of all PV dose groups were significantly increased compared with the H_2_O_2_ group (*p* < 0.01). Among them, the cell viabilities of 10, 30, and 50 μg/mL PV groups under H_2_O_2_ stimulation were significantly increased compared with the control group (*p* < 0.05). Thus, PV had a significant protective effect on H_2_O_2_-induced macrophage. In summary, 10, 30, and 50 μg/mL were selected as low, medium, and high concentrations of PV for subsequent experiments, respectively.

#### 3.3.2. Effect of PV on Intracellular ROS Production

Oxidative stress refers to a state in which reactive oxygen and nitrogen species (RONS) are produced in excess of their own removable range when the organism is subjected to endogenous and exogenous stimuli, resulting in a redox imbalance in the body. Oxidative stress can cause damage to body systems and tissues, resulting in a series of diseases [36]. Fluorescent probes (DCFH-DA) are effective for detecting intracellular ROS production. In this study, the effect of PV on ROS production in RAW264.7 cells was analyzed by detecting the fluorescence intensity of DCF (oxidation product of DCFH-DA). As shown in Figure 3C,D, ROS production was significantly elevated in the H_2_O_2_ group compared to the control group (*p* < 0.01). On the contrary, ROS production was reduced in all three-dose groups of PV compared to the H_2_O_2_ group. Among them, the inhibitory effect of PV in the low-dose group on H_2_O_2_-induced ROS production was not significant (*p* > 0.05), whereas PV in both the medium- and high-dose groups significantly inhibited H_2_O_2_-induced ROS production (*p* < 0.01).

### 3.4. Results of Network Pharmacology Analysis

#### 3.4.1. Screening of Bioactive Components of PV

Detailed information on the chemical constituents of PV from literature queries was obtained from the high-throughput traditional Chinese medicine databases, TCMSP (https://old.tcmsp-e.com/tcmsp.php, accessed on 30 May 2023) and Herb (http://herb.ac.cn/, accessed on 30 May 2023). A total of 17 bioactive components of PV were screened according to the conditions described in Section 2.6.1, and the screening results were shown in Table S1. The action targets of each bioactive components of PV were queried through SwissTarget Prediction database (http://www.swisstargetprediction.ch/, accessed on 12 July 2023) and subsequently analyzed.

#### 3.4.2. Screening of Common Targets Between Bioactive Components of PV and Diseases

**Figure 4:**
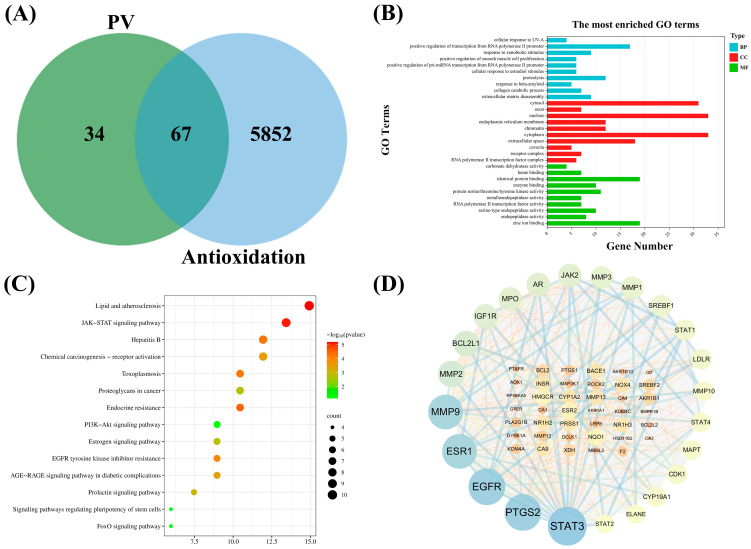
Network pharmacology analysis of antioxidant effect of PV. (**A**) Venn diagram of the intersection of targets of PV with targets of antioxidation. (**B**) Histogram of GO enrichment analysis of antioxidation targets of PV. (**C**) Bubble chart of KEGG pathway enrichment analysis of antioxidation targets of PV. (**D**) PPI network of antioxidant targets of PV, in which the potential targets were represented by nodes, and the interactions between targets were represented by edges. According to the degree of the targets, their colors and sizes were represented from dark to light and from large to small, respectively. The combined scores between targets were indicated by the thickness of edges.

The screened 17 bioactive components of PV were identified by the TCMSP database, and their targets of action were queried by the SwissTarget Prediction database. A total of 101 targets of PV were obtained after deletion of duplicate values. The online tool “jvenn” was used to plot the Venn diagram and to obtain the intersection of 5919 antioxidant-related targets queried in the Genecards database using “antioxidant” as a keyword with 101 targets of PV. As shown in Figure 4A, a total of 67 targets related to the antioxidant effects of PV were finally identified.

#### 3.4.3. Analyses of GO Enrichment and KEGG Pathway Enrichment

The biological processes, cellular components, molecular functions, and signaling pathways involved in the antioxidant effects of PV were obtained by GO enrichment analysis and KEEG pathway analysis. Figure 4B shows the analytical results of GO enrichment, specifically, the antioxidant biological processes (BP) of PV mainly involved a cellular response to UV-A, positive regulation of transcription from RNA polymerase II promoter, response to xenobiotic stimulus, positive regulation of smooth muscle cell proliferation, positive regulation of pri-miRNA transcription from RNA polymerase II promoter, cellular response to estradiol stimulus, proteolysis, response to beta-amyloid, a collagen catabolic process, and extracellular matrix disassembly. The cellular component (CC) enrichment results demonstrated that most of the antioxidant targets of PV were associated with cytosol, axons, the nucleus, the endoplasmic reticulum membrane, chromatin, the cytoplasm, the extracellular space, the caveola, the receptor complex, and the RNA polymerase II transcription factor complex. The significant enrichment terms for PV antioxidant effects in molecular function (MF) included carbonate dehydratase activity, heme binding, identical protein binding, enzyme binding, protein serine/threonine/tyrosine kinase activity, metalloendopeptidase activity, RNA polymerase II transcription factor activity, ligand-activated sequence-specific DNA binding, serine-type endopeptidase activity, endopeptidase activity, and zinc ion binding. Furthermore, the enrichment analysis results of the KEGG pathway based on antioxidant targets of PV are shown in Figure 4C, that is, the pathways related to the antioxidant effects of PV included pathways in cancer, lipid and atherosclerosis, JAK–STAT signaling pathway, hepatitis B, chemical carcinogenesis—receptor activation, toxoplasmosis, proteoglycans in cancer, endocrine resistance, PI3K–Akt signaling pathway, estrogen signaling pathway, EGFR tyrosine kinase inhibitor resistance, AGE-RAGE signaling pathway in diabetic complications, prolactin signaling pathway, signaling pathways regulating pluripotency of stem cells, and the FoxO signaling pathway.

#### 3.4.4. Construction of PPI Network

A string database was used to conduct the protein–protein interaction analysis on the 67 cross-targets of “drug-disease”, and the interaction scores were set to a medium confidence level to construct the PPI network to better elucidate the potential antioxidant mechanisms of PV. Cytoscape software was used to visualize the results of the PPI analysis, and the visualization results are shown in Figure 4D. The composite scores of the interactions between targets ranged from 0.4 to 0.999, which are represented as edges in the figure, with higher composite scores being thicker edges. After comprehensive network pharmacology analysis and topological characterization of the PPI network constructed from 67 targets, BCL2L1, ESR1, JAK2, STAT3, MMP2, and other hub genes related to the antioxidant effects of PV were screened out.

### 3.5. mRNA Expression Levels of Antioxidant-Related Targets of PV

**Figure 5:**
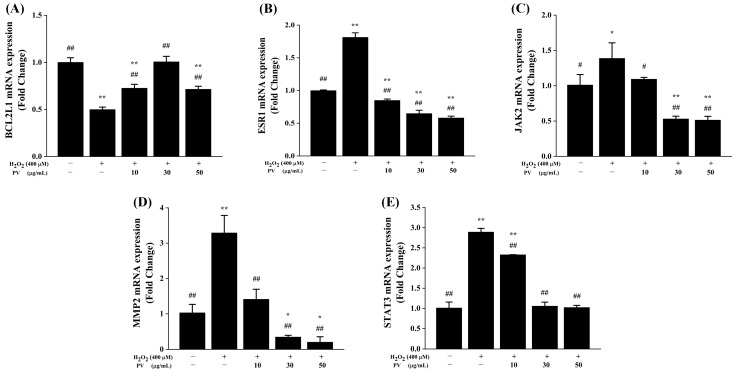
The impact of PV on mRNA expression levels of key antioxidant targets screened through network pharmacology in RAW264.7 cells. The mRNA expression levels of (**A**) BCL2L1, (**B**) ESR1, (**C**) JAK2, (**D**) MMP2, and (**E**) STAT3 were determined by RT-qPCR. The results were expressed as the mean ± standard deviation of three independent experiments. * *p* < 0.05, ** *p* < 0.01, compared with those in the normal control group; # *p* < 0.05, ## *p* < 0.01, compared with those in the model group.

The impact of PV on the mRNA expression levels of key antioxidant targets, which were identified through network pharmacology screening, was assessed using real-time quantitative PCR. The mRNA expression level of BCL2L1 was significantly reduced following H_2_O_2_ (400 μM) stimulation compared to the control group (*p* < 0.01), as depicted in Figure 5A, while the impact of H_2_O_2_ on the mRNA expression level of BCL2L1 was effectively counteracted across all three concentrations of PV (*p* < 0.01). As illustrated in Figure 5B–E, the mRNA expression levels of ESR1, JAK2, MMP2, and STAT3 demonstrated similar tendencies. Namely, in contrast to the control group, H_2_O_2_ stimulation significantly elevated the mRNA expression levels of these four genes (*p* < 0.05), whereas the three concentrations of PV significantly reversed the high expression of their mRNA levels induced by H_2_O_2_ (*p* < 0.05) and presented certain dose-dependent trends.

### 3.6. Protein Expression Levels of Antioxidant-Related Targets of PV

**Figure 6:**
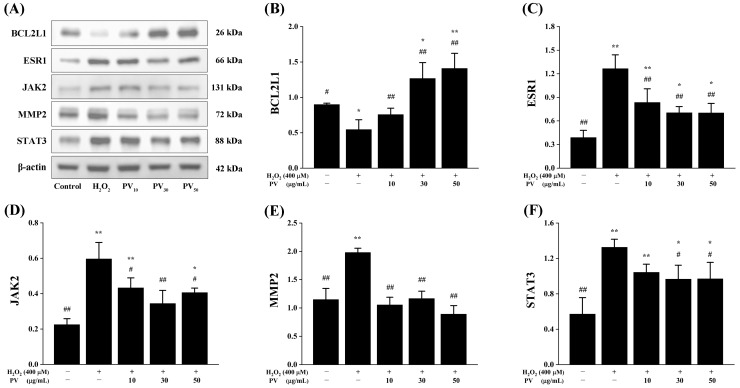
The impact of PV on protein expression levels of key antioxidant targets screened through network pharmacology in RAW264.7 cells. (**A**) Representative protein bands of BCL2L1, ESR1, JAK2, MMP2, STAT3, and β-actin. (**B**) The histograms of bands intensity analysis of (**B**) BCL2L1, (**C**) ESR1, (**D**) JAK2, (**E**) MMP2, and (**F**) STAT3. The results were expressed as the mean ± standard deviation of three independent experiments. * *p* < 0.05, ** *p* < 0.01, compared with those in the normal control group; # *p* < 0.05, ## *p* < 0.01, compared with those in the model group.

In order to further validate the impact of PV on antioxidant targets identified through network pharmacology at the protein level, Western blotting was employed to assess the protein expression levels of the aforementioned five hub genes in various treatment groups. As illustrated in Figure 6, the protein expression levels of BCL2L1, ESR1, JAK2, MMP2, and STAT3 in different treated cells exhibited similar trends to that observed for their mRNA expression levels, respectively. To be specific, the protein expression level of BCL2L1 was significantly reduced after H_2_O_2_ stimulation compared to the control group (*p* < 0.05). However, in a dose-dependent manner, all three concentrations of PV exhibited a significant increase in the protein expression level of BCL2L1 when compared to the H_2_O_2_ group (Figure 6B, *p* < 0.01). In addition, the protein expression levels of ESR1, JAK2, MMP2, and STAT3 in the H_2_O_2_ group exhibited significant increases compared to those in the control group (*p* < 0.01), as depicted in Figure 6C–F. The three concentrations of PV treatments, on the other hand, reduced the high expression of ESR1, JAK2, MMP2, and STAT3 protein levels caused by H_2_O_2_ to different degrees. Among them, compared to the H_2_O_2_ group, the low-dose PV group did not exhibit a significant trend in decreasing STAT3 protein expression level (*p* > 0.05), while the other PV treatment groups significantly reduced the protein expression levels of ESR1, JAK2, MMP2, and STAT3 (*p* < 0.05).

## 4. Discussion

Excessive accumulation of RONS in the body through endogenous and exogenous mechanisms disrupts the redox homeostasis of the organism and causes oxidative stress. Oxidative stress plays a key role in the occurrence and development of various diseases due to its damage to biomolecular and cellular structures, as well as its impact on the functions of organs and normal systems [37]. In particular, oxidative stress is the primary pathogenetic mechanism in diseases such as intestinal ischemia–reperfusion, diseases related to radiation, chemotherapy, and toxicity, whereas in diseases, such as inflammation and diabetes, oxidative stress is a secondary driving force involved in disease progression [38].

In view of its important role in a variety of disease processes, the development of effective antioxidant drugs against oxidative stress is an important and difficult task in the research and development of new drugs. Numerous modern studies have reported that the bioactive constituents of many vegetables, fruits, and traditional Chinese medicines contain a variety of natural antioxidants, such as terpenoids, flavonoids, phenols, saponins, alkaloids, polysaccharides, vitamins, and trace elements [39]. They act directly or indirectly on the antioxidant system of organisms to reduce oxidative stress by removing excess RONS [39,40]. PV is a traditional Tibetan medicine with anti-inflammatory, antioxidant, and antidiarrheal effects documented in the classical works of Tibetan medicine, but its antagonistic effect on oxidative stress and related mechanisms have received extremely limited attention. Qian et al. found that PV containing 21 compounds with antioxidant activity through a new method for rapid screening of antioxidants in natural products [41]. In vitro antioxidant activity of different parts of PV were compared by DPPH method, FRAP method, and other experiments, and it was found that the roots of PV had the strongest antioxidant activity [8]. Consistent with the above findings, this study demonstrated that PV possesses strong in vitro antioxidant activity. Specifically, PV exhibited a strong ability to chelate iron ions and scavenge DPPH radicals, whereas the hydroxyl radical scavenging ability and iron reduction ability were not the key factors affecting the in vitro antioxidant capacity of PV. Therefore, we hypothesized that PV achieves its antioxidant effects by scavenging free radicals directly or by inhibiting the generation of hydroxyl radicals from the Fenton reaction by chelating ferrous ions.

Oxidative stress is one of the important features of gastrointestinal diseases and is associated with the development of many gastrointestinal disorders. It exacerbates the severity of lesions by inducing the deterioration of chronic gastrointestinal diseases and triggering gastrointestinal sequelae [38,42]. It has been reported that ROS induced osmotic diarrhea by participating in intestinal epithelial damage. Meanwhile, ROS increased the intracellular calcium ion concentration, causing cellular chloride secretion and opening of the calcium-activated chloride channel (CaCC), which further induced secretory diarrhea [43]. In addition, oxidative stress has been shown to be involved in the development of diarrhea in rodent models [44], and inhibition of oxidative stress has a beneficial effect in alleviating diarrhea in rodents [45]. Hence, the present study evaluated the in vivo antioxidant effects of PV for the first time using a Senna leaf-induced diarrhea model in rats. The results showed that PV significantly ameliorated the slow body weight gain in rats caused by diarrhea, and the thymus index of rats treated with PV was significantly elevated in a dose-dependent trend. The above experimental results indicated that PV was able to attenuate the effects of oxidative stress on body weight gain and thymus development and improve the antioxidant capacity of rats with Senna-induced diarrhea.

Exogenous or endogenous small molecule antioxidants are not effective in scavenging intracellular ROS, such as O_2_^•−^ or H_2_O_2_. In fact, the antioxidant defense system of organism relies mainly on its own evolved antioxidant enzymes, substrate supply, and damage repair [46]. The most common antioxidant enzymes, such as SOD, CAT, and peroxiredoxins, constitute the organism’s first line of defense against oxidative stress. Non-enzymatic antioxidants such as GSH, inositol, and vitamins, on the other hand, are the organism’s second line of antioxidant defense. While antioxidant enzymes utilize their specific substrates to reduce the production of oxidants that cause direct damage to macromolecules, non-enzymatic antioxidants have the property of scavenging free radicals directly to neutralize the excessive accumulation of ROS [47,48]. Therefore, the basic therapeutic strategy of antioxidant drugs for oxidative stress is to enhance the own antioxidant defenses of organisms, including increasing antioxidant enzymes and their substrates, as well as enhancing the ability to repair oxidative damage. By detecting and analyzing the relevant indexes of antioxidant enzymes in the intestinal tract of Senna-induced diarrhea rats, we found that PV in all three-dose groups increased the activities of the antioxidant enzymes (SOD, CAT) and the content of the non-enzymatic antioxidant (GSH), as well as decreased the content of lipid peroxides (MDA) in the intestines of diarrhea rats to different degrees. In conclusion, PV has strong in vivo antioxidant activity and can enhance the antioxidant defense of rats by increasing the activity of antioxidant enzymes and the content of non-enzymatic antioxidants in the intestinal tract of diarrheic rats, to alleviate the oxidative damage caused by diarrhea to the intestinal tract of rats.

Furthermore, by establishing a model of H_2_O_2_-induced oxidative stress in RAW 264.7 cells, we found that PV at concentrations lower than 200 μg/mL had no toxic effects on cells, and multiple concentrations of PV reversed the adverse effects of H_2_O_2_ on cell viability. In addition, PV significantly inhibited H_2_O_2_-induced cellular ROS production. These results confirmed that PV could alleviate the oxidative damage caused by H_2_O_2_ on cells by inhibiting the excessive accumulation of ROS in the cells, thus, exerting a protective effect against cellular oxidative stress. Systematic network pharmacological analysis, based on large-scale data and bioinformation technology, is an emerging interdisciplinary discipline used to explore the interaction of drug molecules with disease targets [49]. In this study, the mechanism of the antioxidant effect of PV was studied from the perspective of the interaction network between drug molecules and targets, pathways, genes, proteins, and other molecules in organisms using a network pharmacology approach. After analysis, BCL2L1, ESR1, JAK2, MMP2, STAT3, and other hub genes related to the antioxidant effects of PV were screened out.

The B-cell lymphoma-2 (BCL-2) family is a group of regulatory factors closely associated with apoptosis signal transduction, and its member BCL-2 like 1 (BCL2L1, also known as BCL-XL) is found in the mitochondrial membrane, where it is involved in the maintenance of mitochondrial respiratory capacity and affects mitochondrial morphology and cell energy metabolism [50]. It has been reported that oxidative stress regulates the post-translational modification of BCL2L1 and is a key regulator of the synthesis, degradation, and activity of BCL2L1 in cells. Under oxidative stress, the intracellular abundance of functional BCL2L1 is reduced by proteolysis caused by ROS. BCL2L1-mediated multiprotein complex formation is altered due to post-translational phosphorylation regulated by oxidative stress. Among them, BCL2L1, found in the mitochondrial membrane, is involved in the maintenance of mitochondrial respiratory capacity and affects mitochondrial morphology and cellular energy metabolism [50]. It has been reported that oxidative stress regulates the post-translational modification of BCL2L1, which is a key regulator of intracellular BCL2L1 synthesis, degradation, and activity. Under oxidative stress conditions, the intracellular abundance of functional BCL2L1 is reduced by proteolysis caused by ROS [51]. In an in vitro model, H_2_O_2_ treatment has been shown to downregulate BCL2L1 expression at both the mRNA and protein levels [52]. Regulating BCL2L1 increased the survival rate of cells under oxidative stress [53]. Consistent with previous studies, our experiments showed that both mRNA and protein expression levels of BCL2L1 were significantly decreased in H_2_O_2_-treated RAW264.7 cells, whereas PV significantly reversed the inhibition of mRNA and protein expression levels of BCL2L1 by H_2_O_2_, suggesting that PV can protect against oxidative stress by upregulating BCL2L1 in H_2_O_2_-induced macrophages.

Estrogen receptor alpha (ESR1) is one of nuclear estrogen receptors in cells that has regulatory effects on cell growth, proliferation, apoptosis, and maintenance of redox balance [54,55]. ESR1 has been shown to be a major mediator of oxidative stress in cardiomyocytes [56]. In human intrahepatic biliary epithelial cells (HiBECs), ESR1 antagonists were found to protect mitochondria and cells by upregulating mitochondrial membrane potential, reducing cellular ROS production, and inhibiting inflammatory cytokine expression. In contrast, ESR1 agonists caused cellular mitochondrial damage, increased ROS production, and promoted inflammatory cytokine expression [57]. Moreover, it has been reported that naturally derived phytochemicals can counteract the oxidative stress-induced activation of ESR1 to exert a protective effect on cells [58]. For instance, pretreatment with kolaviron can significantly reverse the increased expression of ESR1, the elevated levels of MDA, and pro-inflammatory cytokines, as well as the decreased activities of antioxidant enzymes (GSH-Px, CAT, SOD) induced by 7, 12-dimethylbenzanthracene (DMBA) in rats, thereby alleviating the impact of oxidative stress-related breast cancer in rats [59].

Janus kinase 2 (JAK2) is a non-receptor tyrosine kinase. Studies have indicated that ROS play a significant role in the activation of JAK2. Meanwhile, JAK2 activation has been shown to exacerbate oxidative stress [60]. STAT3 belongs to the signal transducer and activator of transcription (STAT) protein family and is a redox-regulated protein [61]. The intracellular redox environment has a significant impact on the function of STAT3. For example, during the H_2_O_2_-induced oxidative stress, the activity of STAT3 binding to DNA is upregulated, and the addition of ROS scavengers can inhibit the activation of STAT3 [62,63]. As a downstream target gene of JAK2, STAT3 can be activated upon recognition of its SH2 subunit by phosphorylated JAK2 [64]. It has been reported that oxidative stress and stimulation of pro-inflammatory cytokines activate the JAK2/STAT3 pathway, which may also promote oxidative stress and ROS production through feedback effects [65,66]. In the rat model of acute lung injury (ALI), anisodamine suppressed bleomycin-induced inflammation, oxidative stress, and apoptosis in ALI rats by inhibiting the JAK2/STAT3 pathway [67]. Similarly, the natural coumarin analog ryanodine exerts a protective effect on intestinal cells and tight junction proteins by inhibiting oxidative stress, inflammation, and apoptosis through suppression of the JAK2/STAT3 pathway [68].

**Figure 7:**
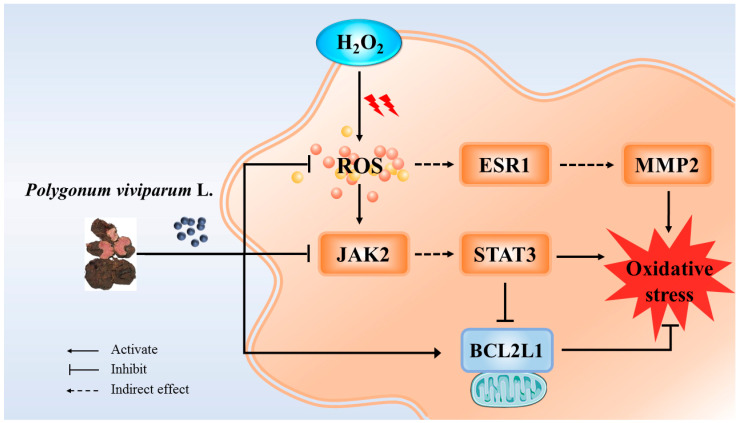
The potential mechanism of PV protects against H_2_O_2_-induced oxidative damage in macrophages.

MMP2 belongs to the family of matrix metalloproteinases (MMPs) and is localized in various intracellular organelles such as cytoskeleton, the nucleus, and mitochondria [69]. Studies on multiple cell models have shown that abnormally elevated ROS levels and oxidative stress promote the expression of MMP2, and the increased MMP2 expression levels in turn exacerbate oxidative stress, inhibit cellular biological functions, and lead to apoptosis, senescence, and DNA damage [70,71,72]. Therefore, MMP2 is considered an effector of oxidative stress and has become one of the important pharmacological targets that have attracted the attention of researchers [69]. In an experimental model of streptozotocin-induced diabetes mellitus (DM) in rats, elevated oxidative stress biomarkers activated MMP2 expression, whereas pretreatment with epigallocatechin gallate nanopreparations significantly reduced plasma MMP2 levels in DM rats and improved antioxidant defenses in DM rats [73]. In the study of lens epithelial cells (LECs) under oxidative stress, MMP2 expression was found to increase with increasing H_2_O_2_ concentration and was significantly inhibited by oxidative stress inhibitors. In addition, interfering with MMP2 expression significantly attenuated the oxidative damage caused by H_2_O_2_ in LECs and mitigated apoptosis and senescence [74]. As mentioned above, a large number of studies have confirmed the close relationship between ESR1, JAK2/STAT3, and MMP2 and oxidative stress. Consistent with the above studies, our study showed that the mRNA and protein expression levels of ESR1, JAK2/STAT3, and MMP2 in macrophages were significantly increased in response to H_2_O_2_ stimulation, but a significant decrease in the mRNA and protein expression levels of these four genes was observed in the PV-treated groups. These results indicated that PV could reduce the level of antioxidant stress in cells and alleviate H_2_O_2_-induced oxidative damage caused by inhibiting ESR1, JAK2/STAT3, and MMP2 (Figure 7).

However, there are several limitations to this study. First, as a natural product, PV has complex active ingredients that require further isolation, purification, and identification. Secondly, the phosphorylation states of these hub genes were not investigated in this study. Whether the phosphorylated hub genes mediate the protective effect of PV on cells under oxidative stress remains to be investigated. In addition, the application of selective inhibitors and overexpression plasmids will help to further investigate the mechanism of action of PV against oxidative stress.

## 5. Conclusions

This study demonstrated that PV, a nature-sourced medicine, has good antioxidant effects through in vivo and in vitro experiments. In addition, activation of BCL2L1 and inhibition of ESR1, JAK2/STAT3, and MMP2 may be the mechanisms by which PV exerts its antioxidant effects. The present study has some limitations, and further work is needed to isolate, purify, and identify the active components of PV, to study the phosphorylation situation of PV antioxidant target genes, and to study and explore the mechanism of PV’s anti-oxidative stress action in greater depth.

## Figures and Tables

**Table 1:** Primers used for qRT-PCR.

Gene Symbol	Accession Number	Sequence (5′ → 3′)
β-actin	11461	(F) TGTTACCAACTGGGACGACA
		(R) GGGGTGTTGAAGGTCTCAAA
MMP2	17390	(F) GCAACGATGGAGGCACGAGTG
		(R) GGGAACTTGATGATGGGCGATGG
STAT3	20848	(F) CGGTTCAGTGAGAGCAGCAAGG
		(R) AGTGAGACAAGAGGAGCAGGTGAG
ESR1	13982	(F) CGCTCTGTGTTGGACTCTGTTAAGG
		(R) ATGGAGATGAAGACAATGGCTGGAAG
JAK2	16452	(F) ATGAGAAGGAGGAGGAGGAGGAG
		(R) TCTGAGGAACTAAGGACAGGTATGC
BCL2L1	12048	(F) TGGTCTTGCTGCTCCTCCTTG
		(R) CTGGTTGCTGTCTCGGTGAATG

## Data Availability

The data used to support the findings of this study are included within the article and the supplementary information files.

## Supplementary Materials

The following supporting information can be downloaded at: https://www.mdpi.com/article/10.3390/antiox14010041/s1, Table S1. The screened active ingredients of PV. Table S2. Results of PPI network analysis.

## Abbreviations

ROS, reactive oxygen species; RNS, reactive nitrogen species; H_2_O_2_, hydrogen peroxide; OH·, hydroxyl radicals; O_2_^−^, superoxide anion; DNA, deoxyribonucleic acid; PV, *Polygonum viviparum* L.; BTW, Baitouweng oral liquid; DMEM, Dulbecco’s modified Eagle’s medium; FBS, fetal bovine serum; CCK-8, cell counting kit-8; Vc, ascorbic acid; DPPH, 2,2-diphenyl-1-picrylhydrazyl; FRAP, ferric reducing antioxidant power; CAT, catalase; GSH, reduced glutathione; MDA, malonaldehyde; SOD, superoxide dismutase; DCFH-DA, dichloro-dihydro-fluorescein diacetate; PPI, protein–protein interaction; GO, gene ontology; BP, biological function/process; CC, cellular component; MF, molecular function; KEGG, Kyoto Encyclopedia of Genes and Genomes; RT-qPCR, real-time quantitative PCR; mRNA, messenger ribonucleic acid; cDNA, complementary DNA; OB, oral bioavailability; DL, drug-likeness; IA, integrated absorbance; RONS, reactive oxygen and nitrogen species; CaCC, calcium-activated chloride channel; BCL-2, B-cell lymphoma-2; BCL2L1, BCL-2 like 1; ESR1, estrogen receptor alpha; HiBECs, human intrahepatic biliary epithelial cells; ESCs, endometrial stromal cells; BPA, bisphenol A; DMBA, 7, 12-dimethylbenzanthracene; JAK, Janus kinase; STAT, signal transducer and activator of transcription; ALI, acute lung injury; MMPs, matrix metalloproteinases; DM, diabetes mellitus; LECs, lens epithelial cells.
